# Protective Effects of *Teucrium Polium* Leaf Ethanolic Extract Against Nicotine-Induced Nephrotoxicity in Mice

**DOI:** 10.33549/physiolres.935314

**Published:** 2025-08-01

**Authors:** Afaf ALATAWI, Saleh MAODAA, Saud ALARIFI, Abdel Halim HARRATH, Esam M. AL-SHAEBI, Dalal A ALHOMOUD, Sarah A. ALAWWAD, Jamaan S. AJAREM

**Affiliations:** 1 Department of Zoology, College of Scie ce, King Saud University, Riyadh, Saudi Arabia; 2 Department of Food Science and Nutrition, College of Food and Agricultural Science, King Saud University, Riyadh, Saudi Arabia

**Keywords:** Nicotine, Teucrium polium, Kidney oxidative stress

## Abstract

This study aimed to examine the protective and antioxidant properties of a *Teucrium polium* leaf extract against acute kidney damage caused by nicotine in male mice. A total of 24 male Swiss albino mice were divided into four groups. The control group (oral solution of 0.9 % NaCl), the positive control group (injections of nicotine at a dosage of 2.5 mg/kg b.w.), the third group (received 100 mg/kg b.w. ethanolic extract of *T. polium*), and the fourth group (nicotine injections at a dosage of 2.5 mg/kg b.w + 100 mg/kg b.w. ethanolic extract of *T. polium*). GC-MS analysis of the plant extract revealed the presence of 16 active compounds. Nicotine administration resulted in a significant increase in kidney biomarkers, namely urea, uric acid, and creatinine, by 50 %, 207 %, and 129 %, respectively, compared to the control group, indicating nephrotoxicity. However, treatment with the *T. polium* extract improved these parameters by 77 %, 79 %, and 83 %, respectively. Furthermore, the nicotine group exhibited elevated levels of nitric oxide (NO) and malondialdehyde (MDA), which are indicators of oxidative stress, as well as decreased levels of glutathione (GSH) and reduced activity of superoxide dismutase (SOD). Conversely, the administration of the *T. polium* extract reversed these effects, suggesting its potential to enhance the antioxidant defense system. This finding was also supported by the improvements observed in the kidney TUNEL assay sections and the preservation of histopathological integrity. In conclusion, the *T. polium* extract demonstrates protective effects against nicotine-induced kidney damage by modulating oxidative stress and antioxidant defense mechanisms.

## Introduction

Tobacco use poses a significant concern for public health and has a detrimental impact on one’s overall well-being. It has been firmly linked to an elevated vulnerability to cardiovascular diseases, lung cancer, and chronic obstructive pulmonary disease (COPD), resulting in a deterioration of overall quality of life [1]. Moreover, cigarette smoking has been identified as a significant contributing factor to the onset of malignancies impacting diverse organs, including the stomach, liver, pancreas, urinary bladder, renal pelvis, and renal cell [2,3]. Nicotine, the primary alkaloid found in tobacco, undergoes metabolic breakdown into different metabolites and is subsequently excreted in the urine [4].

Epidemiological studies consistently highlight smoking as a prevalent risk factor for various diseases, including kidney cancer and renal disorders [5,6]. Nicotine triggered apoptosis in human renal tubular epithelial cells by promoting the generation of reactive oxygen species (ROS) and inducing cell cycle arrest. Moreover, it activated the MAPK and NF-κB signaling pathways [7].

Nicotine and its metabolites are recognized as the most well-established components present in tobacco and tobacco smoke [8]. Recently, researchers have been exploring the effects of nicotine on the advancement of renal diseases [9,10]. The liver predominantly handles nicotine metabolism, the lungs and kidneys also contribute to the metabolic process of nicotine [11]. Furthermore, the mice exposed to nicotine at days 10, 15, and 20 after birth had noticeably lower body weight [11]. The National Institutes of Health (NIH) state that smoking contributes to kidney damage by promoting oxidative stress [12]. Oxidative stress, resulting from an imbalance between the generation of reactive oxygen species (ROS) and the body’s capacity to counteract them, has been implicated in a range of health issues. These include DNA damage, cancer, cardiovascular disease, brain dysfunction, accelerated aging, and various other ailments [13]. Consequently, it becomes imperative to identify strategies aimed at mitigating the oxidative stress in the kidneys resulting from nicotine inhalation [14].

The Lamiaceae family encompasses approximately 210 genera and 3,500 species [15]. Among these species, golden germander, also referred to as *T. polium* holds prominence as one of the well-recognized members. several investigations have been placed into the record that distinct parts of the T. polium plant consist of different biological impacts [15]. It is indigenous to the Middle East and the Mediterranean region. Saligenin, cirsiliol, α- and β-pinene, sabinene, myrcene, germacrene D, limonene, α-caryophyllene, β-caryophyllene and spathulenol represent as chemical constituents present in *T. polium* [15,16,18,20]. Earlier investigations have demonstrated that *T. polium* exhibits pharmacological activities encompassing antibacterial, anti-inflammatory, antioxidant [17], anti-diabetic, anti-inflammatory, and antispasmodic effects [18] and decreases blood pressure [16]. The unsanctioned usage of herbal medicines containing *T. polium* extracts has been linked to numerous cases of hepatotoxicity [16, 17].

The objective of this study was to evaluate the nephroprotective effects of an ethanol extract obtained from *T. polium* leaves in the context of nicotine-induced acute kidney injury in mice. Additionally, the research included assessing improvements in serum indicators of kidney dysfunction and examining the protective and antioxidant capabilities of *T. polium* in alleviating nicotine-induced oxidative stress and kidney damage in mice.

## Material and Methods

### Plant extract

In May 2022, *T. polium* leaves were collected in Al-badyah, Tabuk, Saudi Arabia, located south of Tabuk at coordinates 27°45′59.5″N, 36°31′48.8″E. The plant was identified by the Dr. Rajagopal Rajakrishnan, at the department of Botany and Microbiology, college of Science at King Saud University in Riyadh, Saudi Arabia, and a voucher specimen (No. 24759) from the herbarium. The extraction process of *T. polium* leaves was conducted as previously reported [18] with minor changes. The leaves were ground (PRONEX, China) into a powder after being air-dried. Using ethanol (SIGMA-ALDRICH, CAS No.: 64-17-5, Germany) as the extraction solvent, the dried, powdered plant material was exposed to 72 hours of cold maceration for extraction. Subsequently, it underwent filtration and concentration using a rotary evaporator at 50 °C under reduced pressure. The ethanol extract was gathered, placed in airtight containers, and stored at −20 °C until required.

### Examination of the ethanol extract derived from T. polium leaves through gas chromatography-mass spectrometry (GC-MS)

The ethanolic extract was analyzed for its phytochemical properties using a GC-MS (Agilent Technologies, United States) instrument (Thermo MS DSQ II) from Thermo Scientific Co., specifically the Thermo GC-TRACE ultra ver.: 5.0 model[19]. The GC-MS system was operated under specific experimental conditions, utilizing a TR 5-MS capillary standard non-polar column with dimensions of 30Mts, ID: 0.25 mm, and a film thickness of 0.25μm. The mobile phase (carrier gas: He) maintained a flow rate of 1.0 ml/min. In the gas chromatography phase, a temperature program (oven temperature) initiated at 40 °C and increased to 250 °C at a rate of 5 °C/min, with an injection volume of 1 μl. Samples dissolved in chloroform were subjected to a full scan range of 50–650 m/z, and the obtained results were compared using the Wiley Spectral library search program.

### Experimental animals and housing

Twenty-four Swiss albino male mice were obtained from King Saud University’s College of Science’s animal house. They were kept and observed under strict pathogen-free conditions and weighed 30–35 g when they were 8–10 weeks old. All procedures involving animals in this study followed the guidelines established by the Committee for Control and Supervision of Experiments on Animals (CPCSEA) regarding the care and utilization of experimental animals. The study protocol obtained approval from the Animal Ethics Committee at King Saud University Ethics Agreement ID: (KSU-SE-79-23). Before the commen-cement of the experiment, a one-week acclimatization period was allocated for all animals, during which they were housed in well-ventilated plastic cages. The animals were maintained under standard laboratory conditions, including a temperature of 23 °C, relative humidity ranging from 60 % to 70 %, and a 12-hour light and dark cycle. They received a standard laboratory diet (Crude protein: 20 %, Crude fat 4 %, Crude fiber 3 %, Ash 6 %, Salt 0.50 %, Calcium 1 %, Phosphorus 0.60 %, Vitamin A 20 %, Vitamin D 2.20 %, Vitamin E 70 % and Energy, ME Kcal/kg 2850) as their source of food.

### Chemicals

Pure liquid nicotine was utilized in this experiment; it was acquired from SOMATCO in Riyadh, Saudi Arabia. Nicotine was administered subcutaneously to mice every day.01 ml (2.5mg/kg). In distilled water, nicotine was dissolved.

### Experimental approach

For this investigation, 24 male Swiss albino mice were employed and distributed into four groups, each comprising six mice. Group 1, designated as the negative control, was given drinking water through oral administration and distilled water subcutaneous for a duration of three weeks. Group 2, the positive control group, was administered a daily subcutaneous dose of nicotine at a concentration of 2.5 mg/kg body weight for a duration of three weeks[20]. Group 3, the experimental group, was given a daily dose of the ethanolic extract of *T. polium* leaves at 100 mg/kg body weight for a duration of three weeks [21]. Group 4, in another experimental group, the researchers administered the ethanolic extract of *T. polium* leaves at a dosage of 100 mg/kg body weight to mice. These mice also received daily injections of nicotine at a dose of 2.5 mg/kg body weight for a period of three weeks [21].

### Blood collection

Hematologic specimens (comprising 6 mice per group) will be procured under ketamine/xylazine anesthesia upon completion of the three-week experiment and 24 hours subsequent to the last administered dose [22]. The blood was collected in special tubes (SPINWIN TM Micro Centrifuge Tube 1.5 ml PP Natural, Trasons Cat. No.500010-N, India), after that, spinning the blood samples at a speed of 3000 rpm for 15 minutes, the samples were left at room temperature (22–25 °C) to allow the formation of clots for approximately 30 minutes, the serum was then separated and stored in the refrigerator at −20 °C until the biochemical assays are performed.

### Tissue preparation and histopathological studies

Kidneys were quickly resected and weighed after decapitation and dissection. For histopathological and biochemical examination, tissue samples from the kidney were taken. A tissue sample was removed, fixed for 24 hours in 10 % phosphate-buffered formalin, and then transferred to 70 % alcohol for histology. The tissue sections will be removed and then undergo staining with hematoxylin and eosin (H&E). A Teflon homogenizer was employed to homogenize 0.5 g of tissue in 5 ml of saline (0.9 % NaCl) (manufactured by Glas-Col, Terre Haute, USA). The supernatants obtained from the centrifuged homogenates (speed of 5000 rpm for 15 minutes) will be collected and preserved in a refrigerated environment at −20 °C until they are utilized for the assessment of biochemical parameters related to antioxidant defenses and markers of oxidative stress.

### TUNEL Assay

The kidney sections were cut into 4 μm pieces after being embedded in paraffin and fixed with 4 % paraformaldehyde. A kidney specimen underwent staining utilizing a TUNEL assay kit-HRP-DAB (ab206386; Abcam, Cambridge, UK), following the prescribed guidelines of the manufacturer, to evaluate cellular apoptosis within the renal tissue.

### Assessment of serum parameters indicative of kidney function

The concentrations of urea and creatinine were evaluated using well-established methods, specifically following the protocols outlined by Fabiny and Ertingshausen [23]for creatinine, and by Tabacco et al. [24] for urea. Uric acid levels were determined using a procedure outlined in a previously reported method [25].

### Evaluating the parameters of the antioxidant defense system and oxidative stress

The kidneys were examined for oxidative stress and antioxidant defense parameters using specially prepared chemical reagents in the laboratory. Measurement of lipid peroxidation (LPO) was conducted as previously described [26]. To precipitate proteins, 0.15 mL of 76 % trichloroacetic acid (TCA) was added to 1 mL of the homogenate. Afterward, the separated supernatants were treated with 0.35 mL of the color-enhancing compound known as thiobarbituric acid (TBA) and incubated at 80 °C for 30 minutes. The absorbance at 532 nm is measured. Tetramethoxypropane (1,1,3,3) is used as the benchmark. The quantity of lipid peroxidation products is estimated by the formation of thiobarbituric acid reactive compounds (TBARS). To measure the glutathione (GSH) content, to each microcentrifuge tube containing 0.5 ml TCA, 0.5 ml sample was added, after that the tubes were gently shaken intermittently for 10–15 min. This was followed by centrifugation at 2000 rpm for 5 min at room temperature. Accurately, 0.2 ml of the resulting clear supernatant was taken and mixed with 1.7 ml phosphate buffer in separate test tubes. Then, Elman’s reagent (0.1 ml) was added to each tube. After 5 min, the optical density was measured at 412 nm against a reagent blank ]30[. The assessment of superoxide dismutase (SOD) activity involved inhibiting pyrogallol autooxidation, a process contingent on the presence of superoxide ions. A single unit of enzyme activity is defined as the amount of enzyme that causes a 50 % decrease in extinction changes compared to the control within a period of one minute [28]. Nitric oxide (NO) levels were determined through a colorimetric method. This involved the diazotization of nitrous acid, which, when coupled with sulphanilamide and N-(1-naphthyl) ethylenediamine in an acidic environment, along with the presence of nitrite, produced a brightly reddish-purple azo dye. The resulting dye was detectable at 540 nm [29].

### Statistical analysis

The means ± standard error of the means (SEM) were used to present the data. Statistical analysis was performed using SPSS version 28. To compare different groups, a statistical method called one-way analysis of variance (ANOVA) was used, followed by Tukey’s multiple comparison test. A p-value below 0.05 was used to determine statistical significance (p<0.05).

## Results

### The phytochemical composition of the ethanol extract from T. polium was assessed through gas chromatography-mass spectrometry (GC-MS)

Several compounds were identified when the ethanolic extract of *T. polium* was analyzed using gas chromatography-mass spectrometry (GC-MS). The utilization of GC in combination with mass spectrometry facilitated the identification of these substances. The various compounds identified through gas chromatography-mass spectrometry (GC-MS) in the ethanolic extract of *T. polium* are listed in Table 1. p-Vinylguaiacol, Phenol, 2,6-dimethoxy, 2,4-Di-tert-butylphenol, Spathulenol, Megastigmatrienone, 4,4,5,8-Tetramethylchroman-2-ol, Coniferyl alcohol, Dodeca-namide, n-Hexadecanoic acid, Hexadecanoic acid, ethyl ester, Phytol, Hexadecanamide, Stearic acid, ethyl ester, 9-Octadecenamide, Phenol, 2,2′-methylenebis[6-(1,1-dimethylethyl)-4-methyl, Palmitic acid β-monoglyceride were discerned in the ethanol extracts of *T. polium*.

**Fig. 1:**
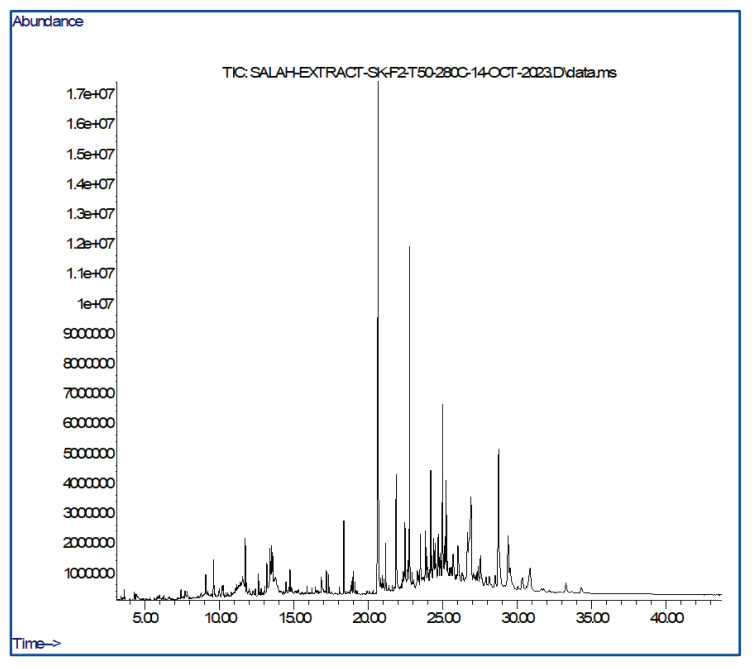
GC-MS of the ethanolic extract of *Teucrium polium* showing 16 peaks with retention times ranging from 9.07 min to 21.86 min.

The GC-MS spectrum, as depicted in Figure 1, confirmed the presence of various components with distinct retention times. The mass spectrometer’s analysis of compounds eluted at different times determines the nature and structure of these compounds. The prominent component undergoes fragmentation, generating peaks at varying m/z ratios. These mass spectra function as unique fingerprints within the data library for each compound. In the current study, the formula and structure of 16 distinct biomolecules were predicted. Further research could potentially result in the discovery and isolation of bioactive compounds. The subsequent analysis of these compounds, including their structural elucidation and pharmacological activity testing holds significant potential for future drug development.

### The impact of T. polium leaf extract treatment on serum parameters associated with kidney function

**Fig. 2:**
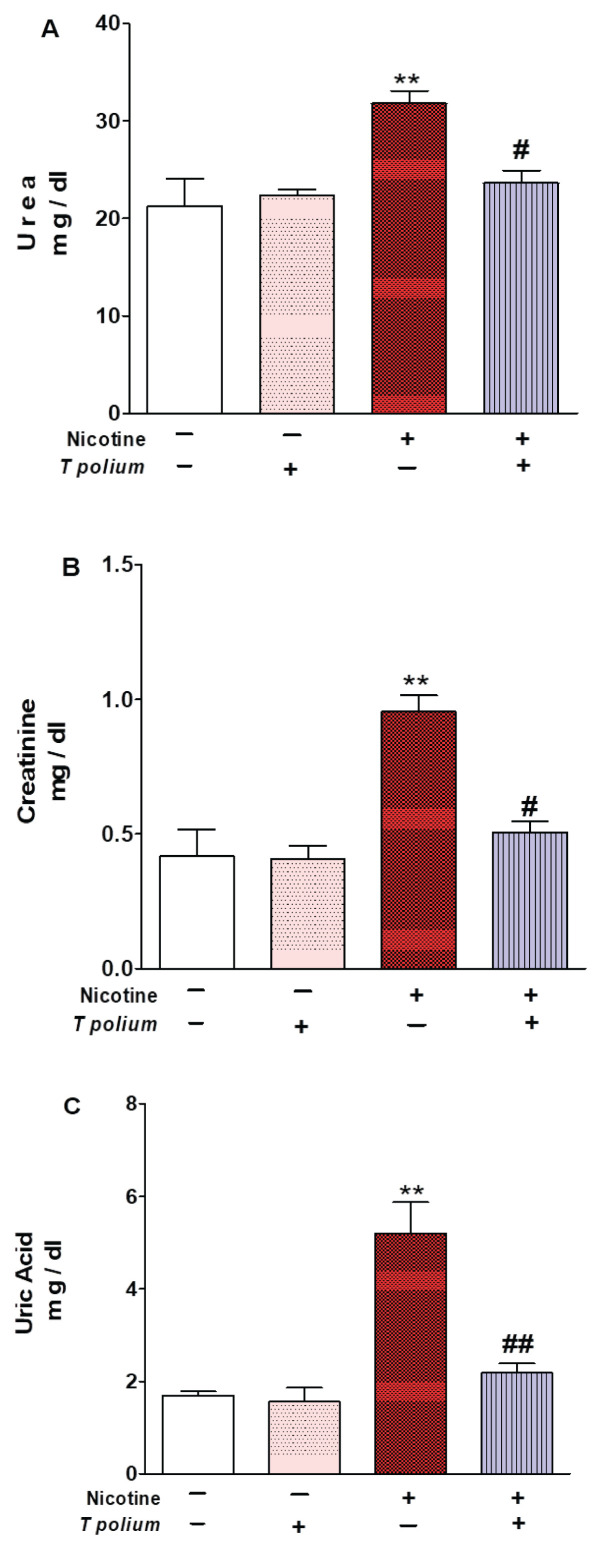
*T. polium* leaves extract ameliorated serum urea (**A**), and creatinine (**B**) levels in nicotine-administred adult mice. Data are presented as mean ± standard error of the mean (SEM). ** p<0.01 versus Control and ^#^p<0.05 versus nicotine. Uric acid level (**C**). Data are presented as mean ± standard error of the mean (SEM). **p<0.01 versus Control and ^##^p<0.01 versus nicotine.

The administration of nicotine subcutaneously at a dosage of 2.5 mg/kg body weight to rats over a three-week daily regimen resulted in a markedly significant elevation (p<0.01) in serum levels of urea, uric acid, and creatinine, compared to the corresponding negative control group. Treatment with the ethanolic extract of *T. polium* leaves exhibited a significant amelioration of heightened creatinine and urea levels (p<0.05) and uric acid levels (p<0.01), demonstrating a highly significant restoration to normal levels in rat models injected with nicotine. These results indicate that the ethanolic extract derived from *T. polium* leaves may have a beneficial effect in guarding against nicotine-induced kidney damage, as demonstrated in Figure 2.

### The effect of administering T. polium leaf extract on markers of kidney oxidative stress and the antioxidant defense system was examined

**Fig. 3:**
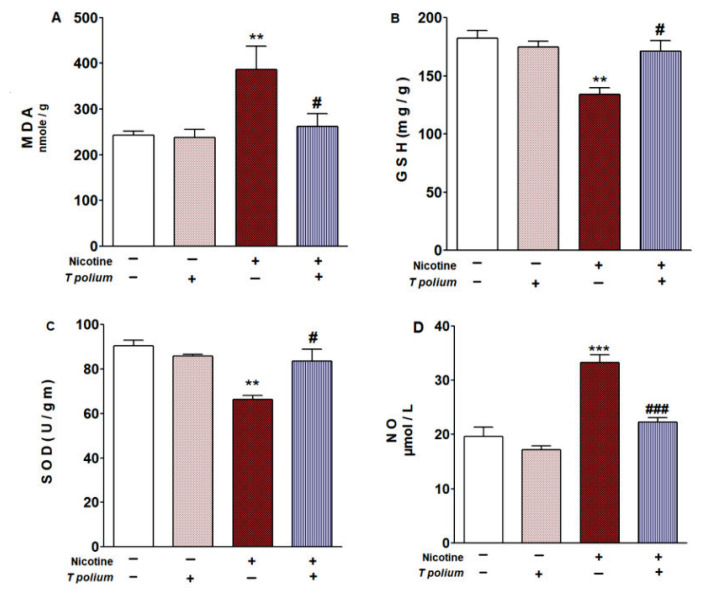
*T. polium* leaves extract decreased LPO (**A**) and NO level (**D**), while increased GSH (**B**), and SOD (**C**) in nicotine-administered adult mice kidney. Data are presented as means ± standard error of the mean (SEM). **p<0.01 and ***p<0.001 versus Control, ^#^p<0.05 and ^###^p<0.001 versus nicotine. Data means ± standard error of the mean (SEM).

Following the administration of nicotine, there was a highly significant (p<0.01) decrease in kidney glutathione (GSH) content and superoxide dismutase (SOD) activities, accompanied by a highly significant (p<0.01) increase in renal lipid peroxidation (LPO) and a very highly significant (p<0.001) elevation in nitric oxide (NO) levels. Rats that received the ethanolic extract derived from *T. polium* leaves followed by subcutaneous nicotine injection, exhibited a significant (p<0.05) reduction in kidney LPO and a very highly significant (p<0.001) decrease in kidney NO levels. Moreover, this treatment significantly (p<0.05) ameliorated the reduced kidney GSH content and SOD activity compared to the controls administered with nicotine (Fig. 3).

### Effect of treatment with T. polium leaves extract on the kidney histopathological changes

**Fig. 4:**
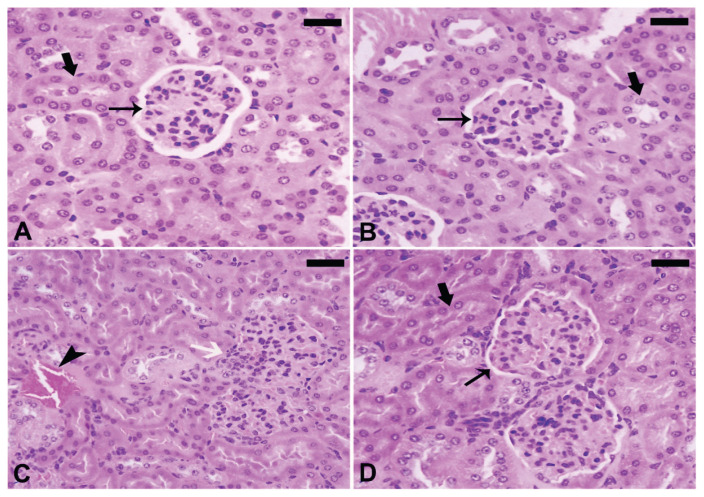
Images of kidney sections stained with hematoxylin and eosin (H&E) were captured using a microscope. (**A**) Represents the negative control group, while (**B**) depicts *T. polium*-treated mice, illustrating Glomerulus (thin arrow) and Renal tubules (thick arrow). (**C**) Displays nicotine-exposed mice with noticeable features including inflammatory cells (White arrow), Hemorrhage between tubules (arrowhead), and degeneration of some renal tubules. (**D**) Shows nicotine-exposed mice treated with *T. polium*, indicating an improved kidney structure. [Scale bar = 50 μm (A, B, C, D)].

Histopathological alterations in the kidney are depicted in Figure 4. The histological sections from normal mice (Fig. 4A) exhibited the typical, undisturbed architectural features. In contrast, mice administered with the ethanolic extract of *T. polium* leaves displayed well-preserved renal tubules (indicated by the thick arrow) and glomeruli (indicated by the thin arrow) (Fig. 4B). Inflammatory cells (White arrow), tubule hemorrhage in between (arrowhead), and partial renal tubule damage were all visible in nicotine-induced mice (Fig. 4C). *T. polium* treatment of nicotine-injected mice resulted in improved kidney histology (Fig. 4D).

### Effect of treatment with T. polium leaves ethanolic extract on TUNEL Assay kidney sections

**Fig. 5:**
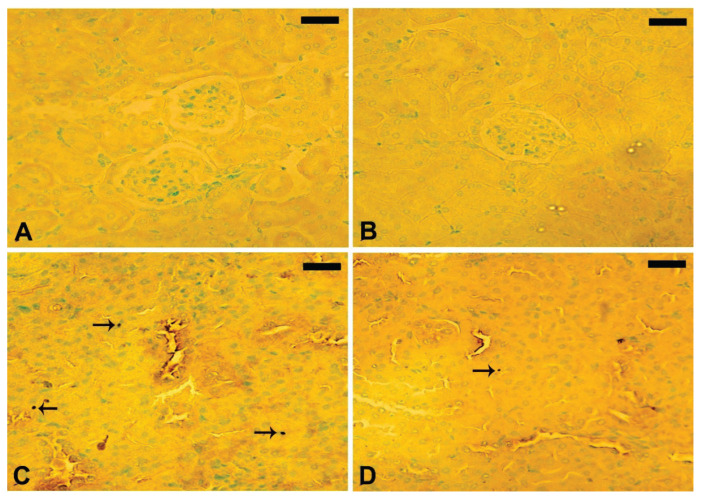
Photomicrographs of TUNEL Assay kidney sections showing *T. polium* -induced apoptotic changes in the kidney of nicotine-injected mice. (**A**) Negative control group. (**B**) *T. polium* treated group. (**C**) Nicotine injected group (**D**) *T. polium* and Nicotine injected group. TUNEL-positive cells appeared brown (arrow). Scale bar =50 μm.

TUNEL Assay changes in the kidneys were illustrated in Figure 5. This figure revealed *T. polium* -induced apoptotic changes in the kidney of nicotine-injected mice. Negative control group (Fig. 5A). *T. polium* treated group (Fig. 5B). Nicotine-injected group (Fig. 5C). *T. polium* and Nicotine injected group (Fig. 5D). TUNEL-positive cells appeared brown.

## Discussion

Nicotine, aside from its well-documented addictive effects, stands out as one of the prevailing contributors to the occurrence of acute kidney injury [5]. Acute kidney injury presents as a sudden decline in kidney function, resulting in the buildup of waste substances like creatinine and urea in the body. This form of injury is frequently associated with additional manifestations, including water and salt retention, a decline in glomerular filtration rate, hyperkalemia, and metabolic acidosis [30].

The kidneys undergo detrimental effects due to the catalyzed biotransformation of nicotine and its reactive metabolites. Consequently, these metabolic processes lead to a rise in the concentration of urea and creatinine in the bloodstream. An increase in the concentration of urea and creatinine in the bloodstream can be seen as a sign that the kidneys are not functioning optimally and are unable to effectively remove toxic metabolic byproducts from the body [31]. In our investigation, male mice subjected to subcutaneous injections of nicotine alone (2.5 mg/kg body weight/day) over a continuous three-week period exhibited markedly elevated serum concentrations of urea, creatinine, and uric acid in comparison to the negative control group, with statistical significance (p<0.01).

These outcomes align with the findings reported by numerous researchers in previous studies [32–35]. Hence, the outcomes of the serum measurements for urea, creatinine, and uric acid in our study signify the potential protective effects associated with the oral administration of *T. polium* leaves ethanolic extract against nicotine-induced kidney damage in mice. Therefore, it can be deduced that the process of isolating and studying the biological activity of phytochemical constituents presents a promising research opportunity. This approach introduces a new perspective to the study of individual components and their effectiveness in pharmacology [19].

The integrity and functionality of cellular processes are reliant on the presence of membrane lipids. The generation of free radicals is expected to cause changes in the structure, fluidity, transport, and antigenic properties of cell membranes. These alterations are crucial in the development of organ disorders, as they lead to the degradation of phospholipids and the occurrence of lipid peroxidation [36]. Antioxidants possess the ability to alleviate the effects of oxidative stress, oxygen-derived free radicals, and the resulting complications [37]. Earlier phytochemical fractionation of *T. polium* leaves extracts has demonstrated the presence of phenolic acids, flavonoids, chlorogenic acid, as well as terpenes and sterols [38,39]. These inherent compounds are characterized by their capacity for radical scavenging, indicating antioxidant efficacy [40]. Our evaluations of oxidative stress parameters revealed a noteworthy reduction in renal malondialdehyde (MDA) and nitric oxide (NO) levels in mice treated with *T. polium* extracts. Conversely, renal superoxide dismutase (SOD) activity and glutathione (GSH) content demonstrated a significant increase compared to the intoxicated group. The antioxidant effect of our extract is primarily ascribed to the presence of diverse bioactive phytoconstituents, facilitating the prevention of lipid peroxidation and scavenging of free radicals. These findings align with previously reported data on the protective efficacy of *T. polium* leaves 40].

A histopathological examination was conducted on all mice, revealing that nicotine administration resulted in pronounced histopathological damage. These findings align with earlier studies [41,42]. Conversely, mice groups treated with *T. polium* leaves ethanolic extract in conjunction with nicotine exhibited amelioration in their kidney tissues, a deduction substantiated by the findings from the TUNEL assay. The adverse impact of nicotine on kidney histopathology was inferred from the outcomes of the histopathological examination.

Administering nicotine subcutaneously at a dosage of 2.5 mg/kg body weight induced severe histopathological damage in the kidneys of the injected mice. Notable changes encompassed degeneration of collecting ducts, congestion, vacuolization in the urothelium, and blood infiltration in the kidney calyx. Additional alterations included edema or infiltration of red blood cells (RBCs) in the interstitial (intertubular) tissues, degenerated kidney tubules, and the presence of sloughed oxidants in the tubular lumen. These observed outcomes align with prior research documenting kidney damage resulting from chronic nicotine administration [43]. In summary, the administration of *T. polium* leaves ethanolic extract notably mitigated renal dysfunctions. The main evidence for this conclusion is the decrease in levels of urea, uric acid, and creatinine in the blood, along with a decrease in renal malondialdehyde (MDA) and nitric oxide (NO) levels. In addition, there is an increase in renal superoxide dismutase (SOD) activity and glutathione (GSH) content. The results from histopathological observations and the TUNEL assay further confirm the protective effects of *T. polium* on the kidneys.

## Conclusion

Our study reveals that the ethanolic extract obtained from *T. polium* leaves exhibits a protective effect against nicotine-induced kidney damage. This beneficial impact is achieved through the reduction of oxidative stress and enhancement of the body’s antioxidant defences, leading to improved kidney function as evidenced by serum levels. Furthermore, we observed an improvement in the structural integrity of kidney tissue and a decrease in programmed cell death. Based on these findings, we recommend further experiments to isolate and assess the efficacy of the active compounds present in the plant extract.

## Figures and Tables

**Table 1:** The determination of the phytochemical composition of the ethanol extract from *T. polium* was conducted through gas chromatography-mass spectrometry (GC-MS).

*N* *^o^*	t_R (min)_	Proposed compound	MW	Formula
***1***	9.07	p-Vinylguaiacol	150	C_9_H_10_O_2_
***2***	9.60	Phenol, 2,6-dimethoxy	154	C_8_H_10_O_3_
***3***	11.73	2,4-Di-tert-butylphenol	206	C_14_H_22_O
***4***	12.61	Spathulenol	220	C_15_H_24_O
***5***	13.18	Megastigmatrienone	190	C_13_H_18_O
***6***	13.61	4,4,5,8-Tetramethylchroman-2-ol	206	C_13_H_18_O_2_
***7***	14.46	Coniferyl alcohol	180	C_10_H_12_O_3_
***8***	14.73	Dodecanamide	199	C_12_H_25_NO
***9***	16.84	n-Hexadecanoic acid	256	C_16_H_32_O_2_
***10***	17.18	Hexadecanoic acid ethyl ester	284	C_18_H_36_O_2_
***11***	18.35	Phytol	296	C_20_H_40_O
***12***	18.97	Hexadecanamide	255	C_16_H_33_NO
***13***	19.11	Stearic acid ethyl ester	312	C_20_H_40_O_2_
***14***	20.66	9-Octadecenamide	281	C_18_H_35_NO
***15***	21.16	Phenol, 2,2′-methylenebis[6-(1,1-dimethyl ethyl)-4-methyl	340	C_23_H_32_O_2_
***16***	21.86	Palmitic acid β-monoglyceride	330	C_19_H_38_O_4_

tR (min)=Retention time

## Abbreviations

GC-MS: gas chromatography-mass spectrometry
LPO: lipid peroxidation
GSH: glutathione
SOD: superoxide dismutase
NO: Nitric oxide
MDA: malondialdehyde
TCA: trichloroacetic acid
ROS: reactive oxygen species
